# Correspondence

**Published:** 1908-04

**Authors:** A. Fleming

**Affiliations:** Atlanta, Ga.


### CORRESPONDENCE

Editor Journal-Record of Medicine,
Atlanta, Ga.

Dear Sir:—

Have just received a copy of the Journal, the first I have seen, and I note what you say in regard to driving charlatans out of the State.

I know that the quacks of all descriptions can be eliminated if each physician will realize that he has a certain amount of this work to do individually. The trouble is, we depend too much on each other and forget that we have something to do ourselves.

I am writing you, not exactly on the line of "quackism," but for humanity's sake as I see it. And you might say this will to some extent eliminate some of it's practices or cut off one of the biggest inroads to patent medicines, etc.

You will find enclosed the bill as drafted by Dr. Izlar and myself last year. This bill was tabled for lack of support, or our Senator, Hon. G. W. Deen, withdrew it because of opposition.

I am informed that when this bill came up in the committee room Drs. Harris and Hardiman opposed it. I feel sure if these doctors only knew the condition which exists in the rural districts they would have been heartily in favor of it.

Now, if those who oppose this bill could only have half the experience the country doctors have had, they would be astounded that something had not already been done before now in behalf of these poor women.

Just think of it. The State of Georgia went crazy last year over prohibition and the bill passed. But when a bill was offered for the protection of the women in the rural districts, where hundreds of them die every year in confinement and blood poison as result of same and on account of witchcrafty, superstitious old ignorant women, it was turned down.

Doctor Editor, I wish that you would figure a little on this and satisfy yourself on these facts. In the month of February I was called to see three cases of Puerperal Convulsions, one of which died. Convulsions all due to a kidney trouble and no physician had seen either of them until convulsions set in. Couple my experience with that of every doctor in the State and see how many deaths you have annually from this cause.

I want to say further that this bill is not intended to make the midwives stand an Anatomical and Obstetrical examination, for this we know they can't do or ever will do, but to make them amenable to law and to teach them things they should know, thereby preventing many deaths and diseased women, which is attributable alone to their ignorance.

We will greatly appreciate any assistance you can give us in behalf of this worthy cause.

Yours fraternally,

A. Fleming.

P. S.—This bill has been approved by the Ware County Medical Society.

A BILL TO REGULATE THE PRACTICE OF MIDWIFERY.

Entitled, An act to regulate the practice of midwifery in this State by others than practicing physicians; to impose certain duties upon them; to provide penalties for the violation of the provisions of this Act; and for other purposes.

Section 1. Be it enacted by the General Assembly of the State of Georgia, and it is hereby enacted by authority of the same, that from and after the passage of this Act, all persons desiring to practice or engage in the art or practice of midwifery in this State, shall first obtain a certificate authorizing them to engage in such practice from the President and Secretary of the local Medical Society or Association of the county of their residence, if such exists in said county; and if there is no such Society or Association in such county, then they shall obtain such certificate from the nearest local Medical Society or Association accessible to them. Such midwife shall thereupon before commencing to practice, register in the office of the Clerk of the Superior Court in which he or she resides in a book to be kept for that purpose, his or her name, age, residence, place of birth, and the name of the Medical Society or Association from which he or she holds a certificate as aforesaid, with the date of same. Such certificate shall be first exhibited to the Clerk who shall be satisfied of its genuineness before the applicant shall be permitted to register. The Clerk shall indorse the word "Registered" upon said certificate, signing and dating the entry, and he shall likewise sign and date the entry of registration made in the registry book, as above provided for. The Clerk shall receive a fee of fifty cents for thus registering a midwife, which shall be paid by the person so registered. Such President and Secretary of the local Medical Society or Association shall be satisfied of the competency and good character of the midwife before granting and issuing the certificate as aforesaid.

Sec. 2. Any such registered midwife who may move from one county to another in this State, shall again register in the office of the Clerk of the Superior Court of the county into which he or she removes for the purpose of residing therein as provided in the preceding section.

Sec. 3. Be it further provided, That upon complaint of any licensed physician, against any registered midwife for incompetency, malpractice, or lack of proper knowledge of the duties of midwifery, such midwife shall be cited to appear before the local Medical Society or Association from which he or she holds a certificate, having first been given five days' written notice of the charge or charges preferred and of the time and place of hearing. The hearing shall be had at such time and place, unless continued for cause, before said Society or Association, five members thereof constituting a quorum, and if a majority of the members trying the matter shall find the midwife guilty of incompetency, malpractice or lack of proper knowledge of the duties of midwifery, as aforesaid, then the certificate theretofore granted to such midwife shall be revoked and taken up and cancelled by said Society or Association and the President and Secretary of the Society or Association shall so certify to the Clerk of the Superior Court, who shall write the words "Certificate Revoked" across the registry of such midwife's name where previously recorded as aforesaid.

Sec. 4. Be it further enacted, That when any child is born attended by a midwife and said child has any affection of the eye or eyes at the time of birth, or such affection shall develop soon thereafter, then it shall be the duty of the attending midwife to report such fact to a regular practicing physician, giving the name of the child's parents and the place where said is to be found.

Sec. 5. For the purposes of this Act, a midwife is hereby defined to be a person, male or female, not a practicing physician, who assists women in child-birth, or practices the obstetric art.

Sec. 6. Be it further enacted, That any person other than a practicing physician, who shall practice midwifery without first being registered as provided by sections one (1) and two (2) of this Act, or who shall fail to make a report as provided by section four (4) of this Act, shall be deemed guilty of a misdemeanor and on conviction shall be punished as provided by section 1039 of Volume 3 of the Code of Georgia of 1895.

Sec. 7. Be it further enacted, That all laws and parts of laws in conflict with this Act, be and the same are hereby repealed.

---

A sinus leading high up in the axilla and discharging a moderately clear fluid may communicate with the shoulder joint or pleura.

*American Journal of Surgery.*

